# Use of genome sequence data in the design and testing of SSR markers for *Phytophthora *species

**DOI:** 10.1186/1471-2164-9-620

**Published:** 2008-12-19

**Authors:** Leonardo Schena, Linda Cardle, David EL Cooke

**Affiliations:** 1 Department of "Gestione dei Sistemi Agrari e Forestali", Mediterranean University of Reggio Calabria, Località Feo di Vito, 89124 Reggio Calabria, Italy; 2 Scottish Crop Research Institute, Invergowrie, Dundee, DD2 5DA, Scotland, UK

## Abstract

**Background:**

Microsatellites or single sequence repeats (SSRs) are a powerful choice of marker in the study of *Phytophthora *population biology, epidemiology, ecology, genetics and evolution. A strategy was tested in which the publicly available unigene datasets extracted from genome sequences of *P. infestans*, *P. sojae *and *P. ramorum *were mined for candidate SSR markers that could be applied to a wide range of *Phytophthora *species.

**Results:**

A first approach, aimed at the identification of polymorphic SSR loci common to many *Phytophthora *species, yielded 171 reliable sequences containing 211 SSRs. Microsatellites were identified from 16 target species representing the breadth of diversity across the genus. Repeat number ranged from 3 to 16 with most having seven repeats or less and four being the most commonly found. Trinucleotide repeats such as (AAG)n, (AGG)n and (AGC)n were the most common followed by pentanucleotide, tetranucleotide and dinucleotide repeats. A second approach was aimed at the identification of useful loci common to a restricted number of species more closely related to *P. sojae *(*P. alni, P. cambivora, P. europaea *and *P. fragariae*). This analysis yielded 10 trinucleotide and 2 tetranucleotide SSRs which were repeated 4, 5 or 6 times.

**Conclusion:**

Key studies on inter- and intra-specific variation of selected microsatellites remain. Despite the screening of conserved gene coding regions, the sequence diversity between species was high and the identification of useful SSR loci applicable to anything other than the most closely related pairs of *Phytophthora *species was challenging. That said, many novel SSR loci for species other than the three 'source species' (*P. infestans*, *P. sojae *and *P. ramorum*) are reported, offering great potential for the investigation of *Phytophthora *populations. In addition to the presence of microsatellites, many of the amplified regions may represent useful molecular marker regions for other studies as they are highly variable and easily amplifiable from different *Phytophthora *species.

## Background

The genus *Phytophthora*, with other Oomycetes, fall within the kingdom Stramenopila, which also includes golden-brown algae, diatoms, and brown algae such as kelp [1-4]. This genus stands out among the plant pathogens since a significant number of the 80 or so described species continue to prove a threat to ecosystem stability and plant productivity on a global scale [5-8]. Despite the importance of *Phytophthora *species, studies of their molecular diversity have been limited by the power of the genetic markers and difficulties in comparing results among laboratories. Accurate studies based on the analysis of mitochondrial and nuclear DNA have resulted in a consensus of the phylogenetic relationships within the genus with a grouping into 10 genetically related clades now accepted [2,3,9]. However, these studies were based on genes commonly conserved within a species and therefore unsuitable to characterize intraspecific variability. Other approaches to study intraspecific variability among *Phytophthora *species including RAPD-PCR and AFLP have proved valuable within a particular study but comparing results from one laboratory to another has always proved challenging with such fingerprinting tools [10-13]. Although microsatellites or simple sequence repeats (SSRs) have been recognised as one of the most powerful choices of markers for molecular ecology they have only relatively recently been exploited in the study of *Phytophthora *populations. SSRs are tandemly repeated motifs of one to six bases which occur frequently and randomly in all eukaryotic genomes although their frequency varies significantly among different organisms [14]. They exhibit a high degree of length polymorphism among related organisms due to stepwise mutations affecting the number of repeat units and leading to polymorphism [14,15]. Dinucleotide repeats account for the majority of microsatellites for many species whereas trinucleotide and hexanucleotide repeats are the most likely repeat classes to appear in coding regions because they do not cause a frameshift [16,17]. Major advantages SSRs include: (i) multiple SSR alleles may be detected at a single locus using a simple PCR-based screen, (ii) SSRs are evenly distributed across the genome, (iii) they are co-dominant, (iv) very small quantities of DNA are required for screening, (v) analysis may be semi-automated, and (vi) results are objective compared to random amplification methods [18].

Microsatellites have been used to investigate genetic structure and reproductive biology of Oomycetes species including *Plasmopara viticola*, *P. cinnamomi*, *P. infestans*, and *P. ramorum *[19-21,23-25]. However, a major limitation to their wider exploitation is the need for prior species-specific marker isolation that requires knowledge of the DNA sequence of the SSR flanking regions to which specific primers have to be designed. Such regions are usually conserved within a species but the likelihood of primers successfully working between species decreases with increasing genetic distance and, in practice, primers are usually developed anew for each species [25,26]. Common methods for the discovery of SSR loci are based on constructing genomic DNA libraries enriched for SSR sequences. These methods were utilised for *P. cinnamomi *and *P. ramorum*, however they are time-consuming, and the specific sequencing of DNA libraries required is expensive [20,25]. Many commercial and academic laboratories specialise in microsatellite isolation services and can provide a set of polymorphic microsatellite loci for a new species in 3–6 months for a cost of approximately USD 1,500 per locus, or USD 10,000 for 10–15 loci [14].

The availability of entire genome sequences for an increasing number of species including *P. infestans *, *P. ramorum *and *P. sojae * have proved novel opportunities to identify and evaluate potential SSR markers identified by computational tools (Abajian, 1994, ) [27,28]. This approach has been utilised to identify SSRs for the study of European and USA populations of *P. ramorum *and for monitoring the genetic variation in populations of *P. infestans *across Europe and worldwide [23,24,29,30].

Recently, Garnica et al. used an *in silico *approach to survey and compare simple sequence repeats (SSRs) in transcript sequences from the genomes of *P. sojae*, *P. ramorum *and *P. infestans *[27]. They also evaluated *in silico *transferability of SSRs among the *Phytophthora *species and found that a proportion (7.5%) of primers could, in theory, be transferred between at least two of the three species. In the present study SSRs from *P. infestans P. sojae *and *P. ramorum *were analysed to identify useful loci common to many *Phytophthora *species (Approach 1) or to a restricted number of species closely related to *P. sojae *(Approach 2). Selected loci were amplified and sequenced from 16 (Approach 1) and 5 (Approach 2) different *Phytophthora *species and a comprehensive SSRs dataset was created.

## Results

### Approach 1 – SSRs for many *Phytophthora *species

**Figure 1:**
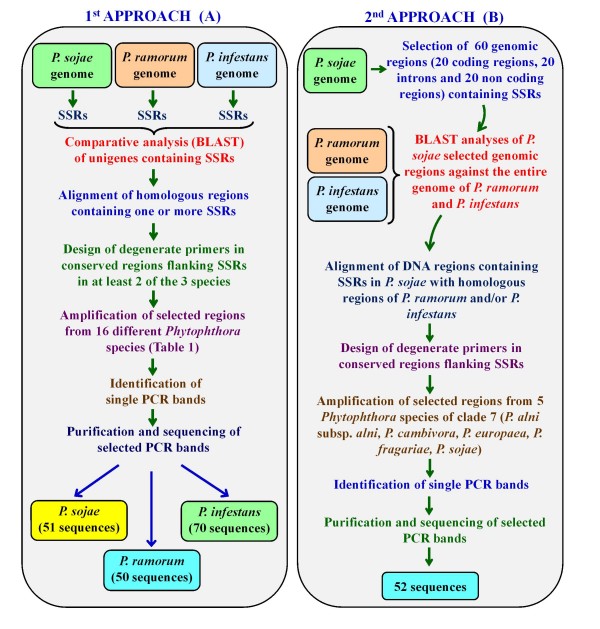
**Schematic representation of two different approaches utilised to identify SSRs in a broad range of *Phytophthora *species (A) or in *Phytophthora *spp. Clade 7 (B)**.

The aim of this approach was to identify loci containing SSRs common to a large number of *Phytophthora *species (Fig. 1A). The method was validated using 16 different species (Table 1) representing the breadth of diversity across the genus [2,3].

**Table 1:** Isolates of *Phytophthora *included in the study, their designations and origins.

*Phytophthora *species	Isolate numbers	Origin		
		
		Host	Country	Year
*P. alni *subsp. *alni*	SCRP2	*Alnus *sp.	UK	1995
	SCRP4^(a)^	*Alnus *sp.	Germany	1995
	SCRP8^(a)^	*Alnus *sp.	France	1996
*P. cambivora*	SCRP67 (IMI 296831)	*Rubus idaeus*	Scotland	1985
	SCRP75^(a)^	*Fagus *sp.	UK	1995
	SCRP80^(a)^	Castanea sativa	Italy	1995
	SCRP82^(a)^	Eucalypt	Australia	
*P. cinnamomi*	SCRP115 (CBS270.55)	*Chamaecyparis lawsoniana*	Netherlands	1993
	SCRP118 (CBS342.72)	*Persea gratissima*	California	1972
	SCRP121^(a)^		Australia	
*P. citricola*	SCRP130	*Rubus idaeus*	Scotland	1986
	SCRP136^(a)^	Soil	UK	1995
	SCRP140^(a)^	*Taxus *sp.	UK	1995
	SCRP143^(a)^	*Quercus robur*	Germany	1994
*P. europaea*	SCRP622	*Quercus robur*	Switzerland	1995
*P. fragariae var. rubi*	SCRP333 (IMI355974)	*Rubus idaeus*	Scotland	1985
*P. ilicis*	SCRP377	*Ilex aquilifolium*	UK	1995
	SCRP379^(a)^	*Ilex aquilifolium*	UK	
*P. infestans*	SC03.26.3.3	*Solanum tuberosum*	Scotland	2003
*P. inundata*	SCRP644 (IMI389751)	*Salix *sp.	UK	1972
*P. lateralis*	SCRP390 (IMI 040503)	*Chamaecyparis lawsoniana*	U.S.A.	1942
*P. nemorosa*	SCRP910			
*P. pseudosyringae*	SCRP674 (IMI390500)	*Malus pumila*	Italy	2001
	SCRP734^(a)^	*Fagus sylvatica*	Italy	2003
*P. psychrophila*	SCRP630	*Quercus ilex*	France	1996
*P. quercina*	SCRP541	*Quercus robur*	Germany	1995
*P. ramorum*	SCRP911	*Rhododendron *sp.	Scotland	2004
*P. sojae*	SCRP555	*Glycine max*	USA	

^(a) ^= Additional isolates utilised to evaluate intraspecific variability

#### Analysis of sequences from *P. infestans*, *P. sojae *and *P. ramorum *genome projects and scanning for homologous SSRs

Predicted gene datasets from *P. infestans **P. sojae *and *P. ramorum * were scanned for the presence of microsatellites defined as short tandem repeat motifs (SSRs) of 2–6 bp. Both perfect and compound SSRs were selected with a minimal acceptable length of 10 bp (dinucelotide motifs) and 12 bp (tri- and tetranucletide motifs). SSRs with a minimum of three repeats were included in the analyses of penta-nucleotide repeats. This search yielded 9333 sequences containing SSRs (1465 from *P. infestans*, 5348 from *P. sojae *and 2520 from *P. ramorum*). The relative abundance of SSRs was 103, 183 and 114 per Mb of predicted gene sequence for *P. infestans*, *P. sojae *and *P. ramorum*, respectively.

Selected regions were compared by BLAST analysis to identify homologous regions flanking SSRs in at least two of the three species (*P. sojae*, *P. ramorum *and *P. infestans*). This analysis identified 4135 SSRs from *P. infestans *(688), *P. ramorum *(1470), and *P. sojae *(1977). A very limited number of loci containing SSRs were common to the three species; most loci were common to *P. ramorum *and *P. sojae *(81.6%), *P. infestans *and *P. ramorum *(7%) or *P. infestans *and *P. sojae *(11%). In most of the cases, homologous loci contained the same SSR motif in different Phytophthoras, however the number of repeats was consistently higher in the 'source' species than the other two.

Among the selected loci, the number of SSR repeats ranged from 3 to 13, from 3 to 12 and from 3 to 17 in *P. infestans, P. ramorum *and *P. sojae *respectively. Most SSRs showed seven repeats or less (98.2% *P. infestans*, 97.4% *P. ramorum*, 94.4% *P. sojae*), with a repeat number of four being the most common in all species.

#### Selection and amplification of target regions containing SSRs

The 4135 homologous regions previously identified were manually analysed to select those with the highest number of repeats and flanked by the most conserved sequences on both sides. The latter condition was necessary to design primers suitable for as many species as possible. Based on this analysis 6, 7 and 12 target regions were identified across the genome of *P. infestans*, *P. ramorum *and *P. sojae *respectively. These regions, containing 8, 17 and 33 SSRs respectively, were selected (Table 2) for amplification from 16 different species of *Phytophthora *representing the breadth of diversity in the genus (Table 1). To this aim, a total number of 62 different degenerate primers (12 for *P. infestans*, 18 for *P. ramorum*, and 32 for *P. sojae*) were designed (Table 2). When target regions contained two or more SSRs and/or were too long to be amplified by a single amplification, a pool of different primers was designed (Table 2). Considerable effort was made to obtain successful amplification from as many species as possible. This involved screening of several primer pairs for each genomic region and for each *Phytophthora *species (Table 2) and adjustment of annealing temperatures and MgCl_2 _concentration for PCR reactions (Tables 3, 4, 5).

**Table 2:** Set of primers designed with the 1^st ^approach (Fig. 1A) to amplify genomic regions with candidate SSRs in a broad range of *Phytophthora *species (Table 1).

	**Forward primers^(a)^**	**Reverse primers^(a)^**	**SSRs^(a)^**	**Source^(b)^**
***P. sojae***	S1F ACGACGTGTCCAAGAACCAC	S3R ATGTTGACCGTGTTCTGCTG	(CCG)_7_;(AGC)_4_; (AGC)_14_	scaffold_3: 1346836–1347814
				
	S4F AARATGACGTGGACKGAGAG	S5R TGATSGTGGAGAARCTCATCT	(AAC)_14_	scaffold_14: 861958–862585
				
	S6F GGAGTTCGCCATCAACAACT	S7R TCAGCTTCTGTCGRTCGAC	(AAG)_14_	scaffold_24: 159790–160289
				
	S8F YGYGTCTCGCCCAYGAC	S9R GACGACACCGGSGAGAG	(ACC)_4_;(AGC)_4_; (AGC)_28_; (AAC)_4_	scaffold_76: 171071–172890
				
	S10F GCGSTACGAGACCTGGAC	S11R GACTCRCCCTTCGACTCSTC	(CAG)_14_	
				
	S12F GGAGGCCGAGTCGGARTA	S13R TAYTCCGACTCGGCCTCC	(AGC)_14_	scaffold_136: 56988–57479
				
	S14F GACGCMSYYGAGTGGAAAG	S15R ATTTKGSACAGATACCGACG	(AAG)_15_	scaffold_65: 332082–332852
				
	S16F TCTACGTGAATGCCATGAGG	S17R CGTTCAGCTTCTGTCGATCR	(AAG)_15_	scaffold_79: 50973–51428
				
	S18F YACCATCTCCAACCTGCTG	S19R CACCACCTCGAGTAGCTCCC	(AGC)_7_; (AGG)_13_	scaffold_2: 1159730–1160958
				
	S19F GGGAGCTACTCGAGGTGGTG	S20R TCGTCTCAATCTCKGACTGA	(AGC)_6_	
				
	S21F ATCTGGGCTTCCASGAGGT	S22R CTGATCCTCCGCCACAY	(AAG)_12_; (ATC)_6_; (ATC)_4_; (AAG)_5_	scaffold_138: 18289–18854
				
	S23F GACTCGGACTCGGACGAC	S25R CTCCTGCTCKTCTTTCAGGC	(AGG)_7_; (AAG)_10_; (GAG)_4_; (AAG)_12_; (GAG)_5_; (AGA)_6_	scaffold_90: 249340–250137
		S37R CTTRCCBTCCTTGTCCTTYT		
				
	S27F GAAGCGCGGGCGWGT	S31R TCCTCCTCTTCTTCTTCGTCW	(AAG)_4_; (AGG)_4_; (ACG)_4_; (AGG)_11_	scaffold_16: 680126–681136
				
	S31F WGACGAAGAAGAAGAGGAGGA	S28R TCATTCATCAGCGTGTCRAT	(GAG)_4_	
	S34F ABGAWGACGABGAGGAVGAV			
				
	S29F MGCAAGAAGGCGTCGTA	S30R CCTTCATCATGAGCTTCTGG	(AGG)_4 _(AAG)_11_	scaffold_40: 353572–354360

***P. ramorum***	R1F GYGGCGGTGGCTACAGYG	R3R CTGCTGYTGCTGGTTGAAAG	(ACC)_4_; (ACC)_5_; (ACC)_4_	scaffold_23: 349447–350425
	R2F CTACTCSAGCCGCTACGC			
				
	R3F CTTTCAACCAGCARCAGCAG	R4R GTTCATCATGCCWCCCATR	(AGC)_8_	scaffold_23: 350406–351828
				
	R4F YATGGGWGGCATGATGAAC	R5R AGGACCAGGAGATGGAGGAC	(AGC)_4_; (AGC)_12_; (AGC)_4_	
				
	R7F TGTTCCARACCCGCTTCC	R8R CACCAAGCAGCACKCGC	(ACG)_9_; (AAC)_5_; (AGC)_10_	scaffold_10: 436897–437690
		R9R GGAACGCACCAAAGACGC		
				
	R10F GGAGATGACGGAAGATGACG	R11R CCATCGAARTACATSACACGA	(AAGCC)_4_; (AGG)_9_; (AAG)_7_	scaffold_5: 750031–750612
				
	R13F AAGTCGAAGCTCGTGGTSAC	R14R GTATCCGCTGRAAGAGCGTC	(AAG)_10_	scaffold_78: 40203–40815
				
	R15F CCGGAGCGCGTGGA	R16R GGTAGTTGAGCGGCTTCTTG	(CCG)_6_	scaffold_2778: 35–305
				
	R16F CAAGAAGCCGCTCAACTACC	R17R TAACGGATCAGCTCTTGCTG	(ATC)_4_; (AGG)_8_	scaffold_2778: 286–1134

***P. infestans***	I3F GCCTGTGGAYGAGAATGGYS	I4R CAGATCCACGACACCRGGY	(AAG)_8_	Pi_002_41652 _Feb05
				
	I5F CATCAACAAGTGCTCGTWCS	I6R TAGTCRAYGTTCTTGTTGTTCA	(AGC)_5_; (AGC)_8_	Supercont1.7 842678–843649
				
	I7F GHGTGGGCGAGTACTCCAAG	I8R AAGCTGGCTATRWACACTGCCG	(AG)_9_	Supercont1.4 1481811–1482015
				
	I9F GCATYGGGTCGTTCCTGTA	I10R AGHGTGCAGTACAGACCCGC	(AAG)_11_	Supercont1.5 1235771–1236143
				
	I11F TCGTCBGTGTCCTCBACGTC	I12R ACCAGCATCTTRTTCTGRGCAG	(ACC)_8_	Supercont1.45 522394–522633
				
	I13F GTCTGCGCTGTCGGAACT	I14R TRATGATGCGGTTCATCTCG	(AAG)_7_; (AAG)_4_	Supercont1.220 167896–168460

^(a) ^= Primers are listed according to the localization in their respective genome projects (*P. sojae*, *P. ramorum *or *P. infestans*) and according to the flanked SSRs. In some circumstances, a pool of different primers was designed to amplify selected genomic regions from as many as possible *Phytophthora *species. Similarly, when target regions contained two or more SSRs and were too long to be amplified by a single amplification a pool of different primers was designed.

^(b) ^= Gene sequences available at  (*P. sojae*),  (*P. ramorum*) and  or  (*P. infestans*).

**Table 3:** Accession numbers and SSRs for GenBank deposited sequences  amplified from 16 *Phytophthora *species (Table 1) using primers designed on *P. sojae *with the first approach (Fig.1A).

***Phytoph***. **species**	**SELECTED PRIMERS^(a)^**											
	
	**S1F-S3R**	**S4F-S5R**	**S6F-S7R**	**S10F-11R**	**S16F-17R**	**S18F-19R**	**S19F-20R**	**S21F-22R**	**S23F-S25R^(1)^**	**^(2)^S31F-28R**	**S27F-S31R**	**S29F-S30R**
***P. alni*** **subsp. *alni*** SCRP2	NS^++^ 58°C/1.7 mM*	EF216617 No SSR 58°C/1.0 mM*	EF216607 (aac)_4_ 58°C/1.7 mM*	EF216602 (agc)_6_ 58°C/1.0 mM*	NS^++^ 55°C/1.7 mM*	NA^+^	EF216590 No SSR 58°C/1.0 mM*	EF216580 (aag)_5_ (agg)_5_ (aag)_4_ (aag)_6_ (agg)_5_ 58°C/1.0 mM*	EF216565 (agg)_7_ (aag)_10_ (agg)_16 _(aag)_12_ (aag)_4_ 58°C/1.0 mM*	NS^++^ 58°C/1.0 mM*	EF216555 (aag)_8_ (agg)_4_ (agg)_4_ (aag)_5_ (agg)_4_ (aag)_4_ (aag)_5_ 58°C/1.0 mM*	EF216552 (aag)_8_ 58°C/1.0 mM*
***P. cambiv***. SCRP67	NS^++^ 58°C/1.7 mM*	EF216618 No SSR 58°C/1.0 mM*	EF216606 (aac)_4_ 58°C/1.7 mM*	EF216601 (agc)_4_ (cg)_5_ 58°C/1.0 mM*	EF216593 No SSR 55°C/1.7 mM*	NS^++^ 58°C/1.0 mM*	NS^++^ 58°C/1.0 mM*	EF216581 (agg)_4_ (aag)_5_ (aag)_4_ 58°C/1.0 mM*	EF216569 (agg)_10 _(aag)_10_ (agg)_9_ (aag)_12_ (agg)_4_ (agg)_7_ 58°C/1.0 mM*	NA^+^	NA^+^	EF216551 (aag)_8_ 58°C/1.0 mM*
***P. cinnam***. SCRP115	NA^+^	NA^+^	NA^+^	NS^++^ 58°C/1.0 mM*	NS^++^ 55°C/1.7 mM*	NA^+^	NA^+^	NS^++^ 58°C/1.0 mM*	NA^+^	NA^+^	NA^+^	EF2165 (aag)_8_ (agg)_5_ (aag)_9_ 58°C/1.0 mM*
***P. citricola*** SCRP130	NA^+^	EF216619 No SSR 58°C/1.0 mM*	NA^+^	NA^+^	NA^+^	NA^+^	NA^+^	NS^++^ 58°C/1.0 mM*	NA^+^	NS^++^ 58°C/1.0 mM*	NA^+^	EF216553 (aag)_7_ (aag)_7_ 58°C/1.0 mM*
***P. europaea*** SCRP622	NS^++^ 58°C/1.0 mM*	EF216616 (acg)_4_ 58°C/1.0 mM*	EF216605 No SSR 58°C/1.7 mM*	EF216600 No SSR 58°C/1.0 mM*	NS^++^ 55°C/1.7 mM*	NS^++^ 55°C/1.7 mM*	EF216589 No SSR 58°C/1.0 mM*	EF216576 (aag)_4_ 58°C/1.0 mM*	EF216568 (agg)_9_ (aag)_10_ (aag)_7_ (agg)_4_ (aag)_6_ (agg)_4_ (agg)_4_ 58°C/1.0 mM*	NA^+^	NA^+^	EF21655054 (aag)_9_ 58°C/1.0 mM*
***P. fragariae*** **var**.***rubi*** SCRP333	NA^+^	NA^+^	NS^++^ 58°C/1.7 mM*	NS^++^ 58°C/1.0 mM*	EF216594 (aac)_4_ 55°C/1.7 mM*	NA^+^	EF216584 No SSR 58°C/1.0 mM*	NA^+^	NA^+^	NA^+^	NS^++^ 60°C/0.7 mM*	EF216542 (aag)_7_ (aag)_8_ 58°C/1.0 mM*
***P. ilicis*** SCRP377	NS^++^ 58°C/1.7 mM*	EF216608 (ccg)_4_ 58°C/1.0 mM*	NA^+^	NS^++^ 58°C/1.0 mM*	NS^++^ 55°C/1.7 mM*	NA^+^	NA^+^	NA^+^	EF216575 No SSR 58°C/1.0 mM*	NA^+^	NS^++^ 60°C/0.7 mM*	EF216543 (aag)_5_ 58°C/1.0 mM*
***P. infestans*** sc 03.26.3.3	NA^+^	EF216615 No SSR 58°C/1.0 mM*	NA^+^	NA^+^	NS^++^ 55°C/1.7 mM*	NA^+^	NA^+^	NA^+^	NA^+^	EF216560 No SSR 58°C/1.0 mM*	NA^+^	NS^++^ 58°C/1.0 mM*
***P. inundata*** SCRP644	EF216624 No SSR 58°C/1.0 mM*	EF216614 No SSR 58°C/1.0 mM*	NA^+^	EF216599 No SSR 58°C/1.0 mM*	NS^++^ 55°C/1.7 mM*	NA^+^	EF216588 No SSR 58°C/1.0 mM*	NA^+^	NA^+^	NS^++^ 58°C/0.7 mM*	NS^++^ 60°C/0.7 mM*	EF216549 (aag)_8_ 58°C/1.0 mM*
***P. lateralis*** SCRP390	NS^++^ 58°C/1.0 mM*	EF216611 No SSR 58°C/1.0 mM*	EF216604 (ac)_5_ 58°C/1.7 mM*	EF216598 (agc)_4_ 58°C/1.0 mM*	NS^++^ 55°C/1.7 mM*	EF216592 (acg)_4_ (agc)_4_ 58°C/1.0 mM*	EF216587 No SSR 58°C/1.0 mM*	EF216579 (aag)_5_ 58°C/1.0 mM*	EF216564 (agg)_5_ (aag)_4_ (aag)_6_ (agg)_4_ 58°C/1.0 mM*	EF216556 (agg)_4_ 58°C/0.7 mM*	NS^++^ 60°C/0.7 mM*	EF216548 (aag)_5_ (aag)_9_ 58°C/1.0 mM*
***P. nemor***. SCRP910	NA^+^	EF216613 (ccg)_4_ 58°C/1.0 mM*	NA^+^	EF216597 No SSR 58°C/1.0 mM*	NA^+^	NS^++^ 55°C/1.7 mM*	NA^+^	NA^+^	EF216572 No SSR 58°C/1.0 mM*	EF216559 No SSR 58°C/1.0 mM*	NA^+^	EF216547 (aag)_5_ 58°C/1.0 mM*
***P. pseudos***. SCRP674	EF216622 No SSR 58°C/1.0 mM*	NS^++^ 58°C/1.0 mM*	NS^++^ 58°C/1.7 mM*	EF216596 No SSR 58°C/1.0 mM*	NA^+^	NA^+^	NA^+^	NA^+^	EF216573 No SSR 58°C/1.0 mM*	EF216557 (agg)_14_ 58°C/1.0 mM*	NS^++^ 60°C/0.7 mM*	EF216546 (aag)_4_ 58°C/1.0 mM*
***P. psychro***. SCRP630	EF216623 (agc)_4_ 58°C/1.0 mM*	EF216612 (ccg)_4_ 58°C/1.0 mM*	NA^+^	NA^+^	NA^+^	NA^+^	NA^+^	NS^++^ 58°C/1.0 mM*	EF216574 No SSR 58°C/1.0 mM*	EF216558 (agg)_6_ (agg)_4_ (aag)_4_ (agg)_4_ 58°C/0.7 mM*	NS^++^ 60°C/0.7 mM*	EF216545 (aag)_4_ 58°C/1.0 mM*
***P. quercina*** SCRP541	EF216621 (agc)_5_ 58°C/1.0 mM*	NA^+^	NA^+^	NA^+^	NA^+^	NA^+^	NA^+^	NS^++^ 58°C/1.0 mM*	EF216563 (aag)_10 _(aag)_5_ 58°C/1.0 mM*	EF216561 (agg)_4_ 58°C/1.0 mM*	NA^+^	EF216544 (aag)_7_ 58°C/1.0 mM*
***P. ramorum*** SCRP911	EF216620 No SSR 58°C/1.0 mM*	EF216610 (acc)_4_ 58°C/1.0 mM*	EF216603 No SSR 55°C/1.7 mM*	NS^++^ 58°C/1.0 mM*	EF216595 No SSR 55°C/1.7 mM*	EF216591 No SSR 58°C/1.0 mM*	EF216586 No SSR 58°C/1.0 mM*	EF216578 (aag)_4_ (atc)_4_ (aag)_5_ (aag)_5_ 58°C/1.0 mM*	EF216562 (aag)_4_ (agg)_4_ (aag)_7_ 58°C/1.0 mM*	NS^++^ 58°C/0.7 mM*	NA^+^	EF216540 (aag)_4_ 58°C/1.0 mM*
***P. sojae*** SCRP555	NA^+^	EF216609 (aac)_14_ 58°C/1.0 mM*	NS^++^ 58°C/1.7 mM*	NS^++^ 58°C/1.0 mM*	EF382779 No SSR 55°C/1.7 mM*	NA^+^	EF216585 (agc)_6_ 58°C/1.0 mM*	EF216577 (aag)_12_ (atc)_6_ (atc)_4_ (aag)_5_ 58°C/1.0 mM*	NS^++^ 58°C/0.7 mM*	NS^++^ 58°C/0.7 mM*	NA^+^	EF216541 (agg)_4_ (aag)_11_ 58°C/1.0 mM*

^(a) ^= Primers listed in Table 2 and not reported in this table are those that did not produce any reliable sequence (see text).

^(1) ^= Primer S25R was replaced by primer S37R for *P. nemorosa, P. pseudosyringae, P. psychrophila*, and *P. ilicis*.

^(2) ^= Primer S31F was replaced by primer S34F for *P. alni, P. citricola, P. infestans, P. nemorosa*, and *P. quercina*.

NA^+ ^= Isolate-primer combinations that did not produce any amplification or produced complex profiles (two or more fragments).

NS^++ ^= Isolate-primer combinations for which single PCR bands were obtained but direct sequencing did not produce reliable results.

°C/mM* = Selected annealing temperature (°C) and MgCl_2 _concentration (mM) in PCR reactions.

**Table 4:** Accession numbers and SSRs for GenBank deposited sequences  amplified from 16 *Phytophthora *species (Table 1) using primers designed on *P. ramorum *with the first approach (Fig. 1A).

***Phytophthora*** **species**	**SELECTED PRIMERS^(a)^**						
	
	^(1)^**R1F-R3R**	**R3F-R4R**	**R4F-R5R**	**R7F-9R^(2)^**	**R10F-R11R**	**R13F-R14R**	**R16F-R17R**
***P. alni *subsp. *alni*** SCRP2	NA^+^	NA^+^	EF216645 No SSR 58°C/1.0 mM*	EF216671 No SSR 58°C/1.0 mM*	EF216662 No SSR 58°C/1.7 mM*	EF216651 (aag)_4_ 58°C/1.7 mM*	EF216625 (acg)_4_ 58°C/1.0 mM*
***P. cambivora*** SCRP67	NS^++^ 58°C/1.0 mM*	NA^+^	NS^++^ 58°C/1.0 mM*	EF216673 (agc)_8_ 58°C/1.0 mM*	EF216663 (agg)_4_ (aag)_4_ (agg)_4_ 58°C/1.7 mM*	NS^++^ 58°C/1.7 mM*	EF216626 (acg)_4_ 58°C/1.0 mM*
***P. cinnamomi*** SCRP115	NA^+^	NA^+^	NA^+^	NA^+^	NA^+^	NS^++^ 58°C/ 1.7 mM*	NA^+^
***P. citricola*** SCRP130	NA^+^	NA^+^	EF216644 No SSR 58°C/1.0 mM*	NA^+^	NA^+^	NA^+^	EF216630 No SSR 58°C/1.0 mM*
***P. europaea*** SCRP622	NS^++^ 58°C/1.0 mM*	NA^+^	EF216643 No SSR 58°C/1.0 mM*	NA^+^	EF216661 (agg)_4_ (aag)_7_ (agg)_5_ 58°C/1.7 mM*	EF216655 (aag)_4_ 58°C/1.7 mM*	NA^+^
***P. fragariae*** ***var. rubi*** SCRP333	NS^++^ 58°C/1.0 mM*	NS^++^ 58°C/1 mM**	EF216634 No SSR 58°C/1.0 mM*	EF216672 (agc)_7_ 58°C/1.0 mM*	EF216657 (aag)_4_ (agg)_4_ 58°C/1.7 mM*	EF216647 (aag)_4_ 58°C/1.7 mM*	NS^++^ 58°C/1.0 mM*
***P. ilicis*** SCRP377	NS^++^ 58°C/1.0 mM*	EF216646 (agc)_4_ (agc)_4_ 58°C/1.0 mM*	EF216635 (agc)_4_ (accat)_5_ 58°C/1.0 mM*	EF216664 (actg)_3_ (agc)_6_ 58°C/1.0 mM*	NS^++^ 58°C/1.7 mM*	EF216648 No SSR 58°C/1.7 mM*	NS^++^ 58°C/1.0 mM*
***P. infestans*** sc 03.26.3.3	NS^++^ 58°C/ 1.0 mM*	NA^+^	NA^+^	NA^+^	NS^++^ 58°C/1.7 mM*	NA^+^	NA^+^
***P. inundata*** SCRP644	EF216633 No SSR 58°C/ 1.0 mM*	NA^+^	NA^+^	NA^+^	NA^+^	EF216654 No SSR 58°C/1.7 mM*	EF216629 No SSR 58°C/1.0 mM*
***P. lateralis*** SCRP390	EF216631 No SSR 58°C/1.0 mM*	NA^+^	EF216642 (aac)_5_ (agc)_5_ 58°C/1.7 mM*	EF216669 (agc)_5_ (aac)_6_ 58°C/1.0 mM*	EF216660 (agg)_4_ 58°C/1.7 mM*	NA^+^	EF216628 (agg)_8_ (agc)_4_ 58°C/1.0 mM*
***P. nemorosa*** SCRP910	NS^++^ 58°C/1.0 mM*	NA^+^	EF216639 (agc)_4_ (accat)_4_ 58°C/1.0 mM*	EF216668 (actg)_3_ (agc)_6_ 58°C/1.7 mM*	NS++ 58°C/1.7 mM*	EF216653 (agg)_9_ 58°C/1.7 mM*	NA^+^
***P. pseudosyringae*** SCRP674	EF216632 No SSR 58°C/1.0 mM*	NA^+^	EF216641 (accat)_4_ 58°C/1.0 mM*	EF216667 No SSR 58°C/1.0 mM*	NA+	EF216652 No SSR 58°C/1.7 mM*	NA^+^
***P. psychrophila*** SCRP630	NS^++^ 58°C/1.0 mM*	NA^+^	EF216640 (accat)_4_ 58°C/1.0 mM*	EF216666 (actg)_3_ (agc)_6_ 58°C/1.0 mM*	NA^+^	NS^++^ 58°C/1.7 mM*	NS^++^ 58°C/1.0 mM*
***P. quercina*** SCRP541	NS^++^ 58°C/1.0 mM*	NA^+^	EF216638 No SSR 58°C/1.0 mM*	EF216674 No SSR 58°C/1.0 mM*	NA^+^	EF216651 (aag)_8_ (aag)_4_ 58°C/1.7 mM*	NA^+^
***P. ramorum*** SCRP911	NS^++^ 58°C/1 mM*	NA^+^	EF216637 (agc)_4_ (agc)_10_ (agc)_4_ 58°C/1.0 mM*	EF216665 (agc)_24_ 58°C/1.0 mM*	EF216659 (aagcc)_4_ (agg)_9_ (aag)_7_ 58°C/1.7 mM*	EF216650 (aag)_10_ 58°C/1.7 mM*	EF216627 (atc)_4_ (agg)_8_ 58°C/1.0 mM*
***P. sojae*** SCRP555	NA^+^	NA^+^	EF216636 (agc)_5_ 58°C/1.0 mM*	EF216670 (agc)_10_ (agcg)_5_ 58°C/1.0 mM*	EF216658 (agg)_5_ (aag)_6_ (agg)_4_ 58°C/1.7 mM*	EF216649 (acg)_4_ (aag)_4_ 58°C/1.7 mM*	NA^+^

^(a) ^= Primers listed in Table 2 and not reported in this table are those that did not produce any reliable sequence (see text).

^(1) ^= Primer R1F was replaced by primer R2F for *P. cambivora*, *P. inundata, P. nemorosa, P. ilicis *and *P. fragaria*.

^(2) ^= Primer R9R was replaced by primer R8R for *P. quercina*.

NA^+ ^= Isolate-primer combinations that did not produce any amplification or produced complex profiles (two or more fragments).

NS^++ ^= Isolate-primer combinations for which single PCR bands were obtained but direct sequencing did not produce reliable results.

°C/mM* = Selected annealing temperature (°C) and MgCl_2 _concentration (mM) in PCR reactions.

**Table 5:** Accession numbers and SSRs for GenBank deposited sequences  amplified from 16 *Phytophthora *species (Table 1) using primers designed on *P. infestans *with the first approach (Fig. 1A).

***Phytophthora*** **species**	**SELECTED PRIMERS^(a)^**					
	
	**I3F-4R**	**I5F-I6R**	**I7F-I8R**	**I9F-I10R**	**I11F-I12R**	**I13F-I14R**
***P. alni *subsp. *alni*** SCRP2	NS^++^ 58°C/1.7 mM*	EF216535 No SSR; 58°C/1.0 mM*	NS^++^ 58°C/1.7 mM*	EF216513 (aag)_4_ (agg)_6_ 58°C/1.0 mM*	NA^+^	EF216477 (agg)_4_ (aag)_5_ 58°C/1.7 mM*
***P. cambivora*** SCRP67	NS^++^ 58°C/1.7 mM*	NA^+^	NS^++^ 58°C/1.7 mM*	EF216516 (acg)_4_ (aag)_5_ (agg)_6_ 58°C/1.0 mM*	NS^++^ 58°C/1.7 mM*	EF216478 (agg)_5_ (aag)_5_ 58°C 1.7 mM*
***P. cinnamomi*** SCRP115	NS^++^ 58°C/1.7 mM*	NS^++^ 58°C/1.7 mM*	NS^++^ 58°C/1.7 mM*	EF216509 (aag)_14_ 58°C/1.0 mM*	EF216494 (aag)_4_ 58°C/1.7 mM*	NS^++^ 58°C/1.7 mM*
***P. citricola*** SCRP130	NS^++^ 58°C/1.7 mM*	EF216534 (agc)_4_; (agc)_5_ 58°C/1.0 mM*	NS^++^ 58°C/1.7 mM*	EF216520 (aag)_4_ 58°C/1.0 mM*	EF216498 (aag)_4_ 58°C/1.7 mM*	EF216482 (agg)_9_ (aag)_5_ 58°C/1.7 mM
***P. europaea*** SCRP622	NS^++^ 58°C/1.7 mM*	NS^++^ 58°C/1.0 mM*	NS^++^ 58°C/1.7 mM*	EF216512 No SSR 58°C/1.0 mM*	NS^++^ 58°C/1.7 mM*	EF216476 (agg)_5_ (aag)_5_ 58°C/1.7 mM*
***P. fragariae*** **var. *rubi*** SCRP333	NS^++^ 58°C/1.7 mM*	NA^+^	NA^+^	EF216500 (acg)_4_ 58°C/1.0 mM*	NA^+^	NA^+^
***P. ilicis*** SCRP377	NS^++^ 58°C/1.7 mM*	EF216532 (agc)_9_ 58°C/1.0 mM*	NA^+^	EF216501 No SSR 58°C/1.0 mM*	EF216495 No SSR 58°C/1.7 mM*	EF216483 (aag)_4_ (agc)_4_ 58°C/1.7 mM
***P. infestans*** sc 03.26.3.3	NS^++^ 58°C 1.7 mM*	EF216524 (agc)_6_ (agc)_5_ 58°C/1.0 mM	NS^++^ 58°C/1.7 mM*	EF216499 (aag)_11_ 58°C/1.0 mM*	EF216487 (acc)_8_ 58°C/1.7 mM*	EF216474 (aag)_7_ (aag)_4_ 58°C/1.7 mM
***P. inundata*** SCRP644	NS^++^ 58°C/1.7 mM*	NS^++^ 58°C/1.0 mM*	NS^++^ 58°C/1.7 mM*	EF216508 No SSR 58°C/1.0 mM*	EF216497 No SSR 58°C/1.7 mM*	NA^+^
***P. lateralis*** SCRP390	NS^++^ 58°C/1.7 mM*	EF216527 (agc)_4_ 58°C/1.0 mM*	NS^++^ 58°C/1.7 mM*	EF216507 No SSR 58°C/1.0 mM*	EF216493 No SSR 58°C/1.7 mM*	EF216481 (agg)_5_ (aag)_4_ 58°C/1.7 mM*
***P. nemorosa*** SCRP910	NS^++^ 58°C/1.7 mM*	EF216531 (agc)_7_ 58°C/1.0 mM*	NA^+^	EF216503 No SSR 58°C/1.0 mM*	EF216492 (aag)_4_ 58°C/1.7 mM*	EF216486 (acg)_4_ (aag)_4_ (agc)_4_ 58°C/1.7 mM*
***P. pseudosyringae*** SCRP674	NS^++^ 58°C/1.7 mM*	EF216529 (agc)_7_ 58°C/1.0 mM*	NS^++^ 58°C/1.7 mM*	EF216502 No SSR 58°C/1.0 mM*	EF216496 (aag)_5_ 58°C/1.7 mM*	EF216485 (acg)_4_ (aag)_4_ 58°C/1.7 mM*
***P. psychrophila*** SCRP630	NS^++^ 58°C/1.7 mM*	EF216528 (agc)_4_ 58°C/1.0 mM*	NS^++^ 58°C/1.7 mM*	NA^+^	EF216491 (aag)_4_ 58°C/1.7 mM*	EF216484 (aag)_4_ (agc)_4_ 58°C/1.7 mM*
***P. quercina*** SCRP541	NS^++^ 58°C/1.7 mM*	NS^++^ 58°C/1.0 mM*	NS^++^ 58°C/1.7 mM*	EF216506 No SSR 58°C/1.0 mM*	EF216490 (aag)_5_ 58°C/1.7 mM*	EF216480 (agg)_6_ (aag)_4_ 58°C/1.7 mM*
***P. ramorum*** SCRP911	NS^++^ 58°C/1.7 mM*	EF216525 No SSR 58°C/1.0 mM*	NS^++^ 58°C/1.7 mM*	EF216505 No SSR 58°C/1.0 mM*	EF216489 (acc)_4_ 58°C/1.7 mM*	EF216479 (aac)_7_ (agg)_9_ 58°C/1.7 mM
***P. sojae*** SCRP555	NS^++^ 58°C/1.7 mM*	EF216526 No SSR 58°C/1.0 mM*	NA^+^	EF216504 (agc)_4_ 58°C/1.0 mM*	EF216488 No SSR 58°C/1.7 mM*	EF216475 (agg)_7_ (aag)_5_ 58°C/1.7 mM*

^(a) ^= Primers listed in Table 2 and not reported in this table are those that did not produce any reliable sequence (see text).

NA^+ ^= Isolate-primer combinations that did not produce any amplification or produced complex profiles (two or more fragments).

NS^++ ^= Isolate-primer combinations for which single PCR bands were obtained but direct sequencing did not produce reliable results.

°C/mM* = Selected annealing temperature (°C) and MgCl_2 _concentration (mM) in PCR reactions.

**Figure 2:**
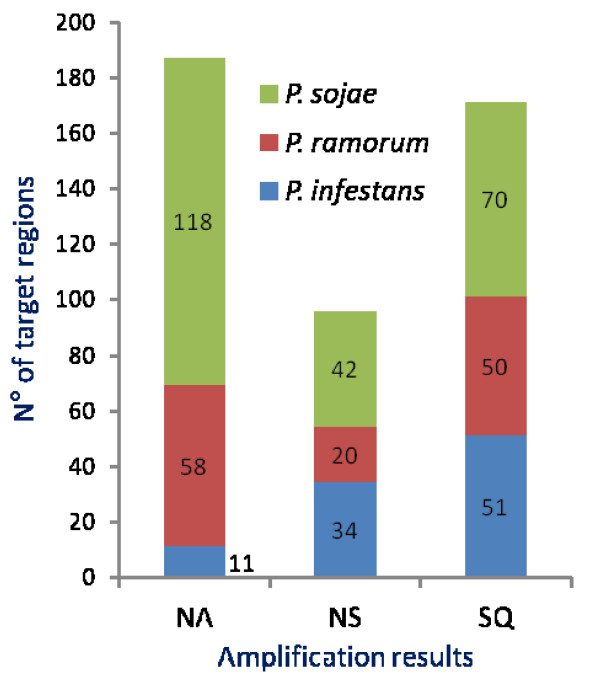
**Amplification results obtained with 16 *Phytophthora *species (Table **1**) using primers designed against *P. sojae, P. ramorum *and *P. infestans *genomes using Approach 1 (Fig. **1A**)**. NA represents primer-species combinations in which amplification reactions did not produce any product or produced complex profiles (two or more PCR fragments) preventing direct sequencing. NS represents primer-species combinations in which amplification reactions produced single PCR bands, however direct sequencing did not yield reliable sequences. SQ represents primer-species combinations in which reliable sequences were obtained.

The resultant primers enabled the amplification of 271 single PCR bands of the expected size (Fig. 2). In the remaining primer-species combinations, 193 amplifications did not produce any product or produced complex profiles (two or more PCR fragments) impeding direct sequencing (Fig. 2). Some primer combinations failed to amplify a product from any of the *Phytophthora *species whereas other combinations amplified single bands from all or most *Phytophthora *species (Tables 3, 4, 5).

#### Sequencing of single PCR bands and scanning for SSRs

**Figure 3:**
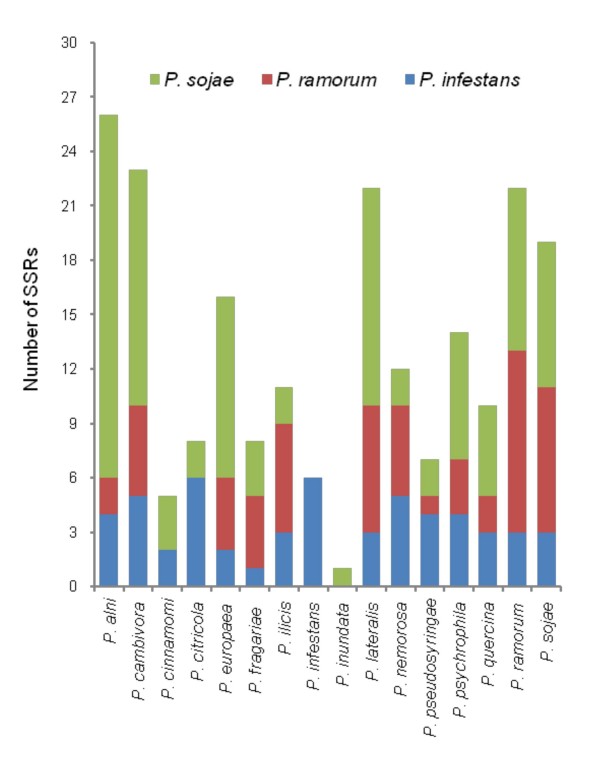
**Number of SSRs identified for each of the 16 *Phytophthora *species using primers designed against *P. sojae, P. ramorum *and *P. infestans *genomes (Approach 1, Fig. **1A**)**.

**Figure 4:**
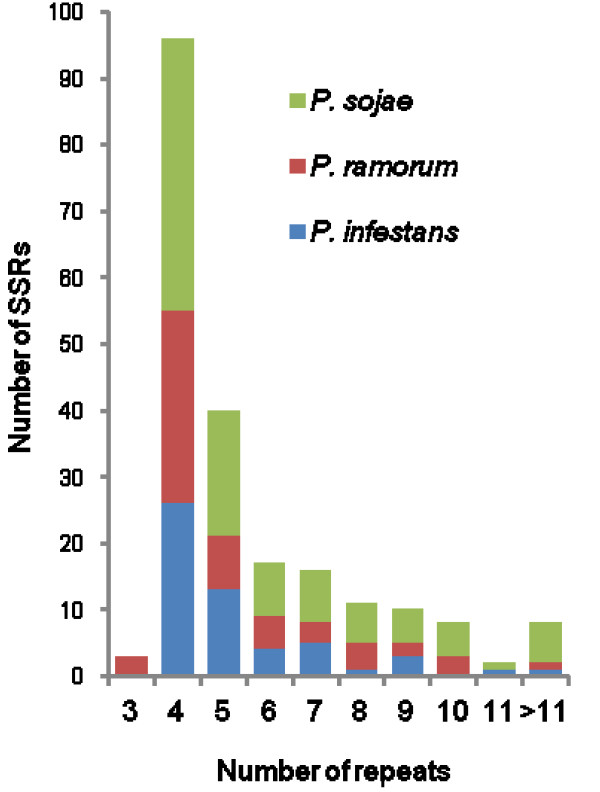
**Number of repeated motifs identified in 16 target *Phytophthora *species (Table **1**) using primers designed against *P. sojae, P. ramorum *and *P. infestans *genomes according to Approach 1 (Fig. **1A**)**.

**Figure 5:**
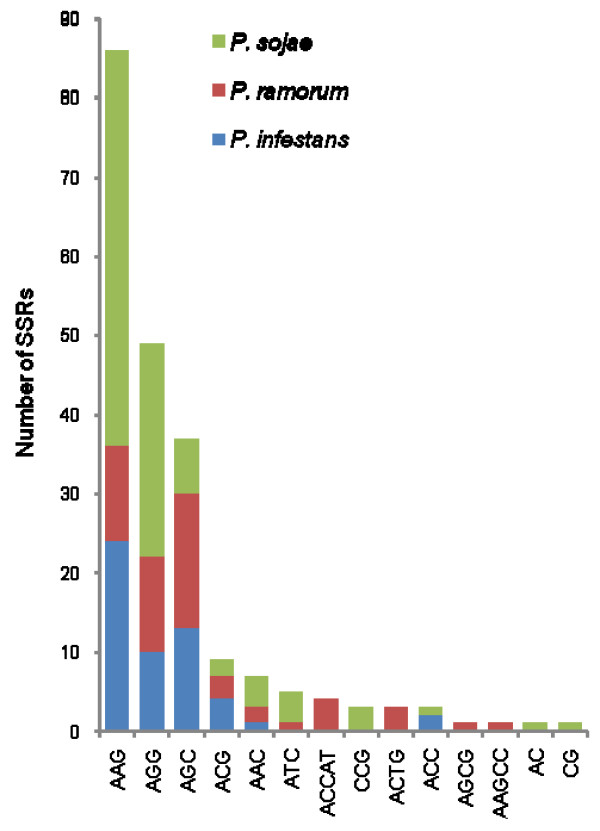
**List and frequency of the different SSR motifs identified in 16 *Phytophthora *species (Table **1**) using primers designed on *P. sojae, P. ramorum *and *P. infestans *genomes according to Approach 1 (Fig. **1A**).**

All single PCR bands (271) were purified to remove excess primers and nucleotides and sequenced in both directions using the same primers used for the amplification. When forward and/or reverse sequences were not identical, amplification, purification and sequencing were repeated twice and all unreliable sequences were discarded. Finally, 171 sequences were obtained with primers designed against *P. sojae *(70), *P. ramorum *(50) and *P. infestans *(51) genomes (Fig. 2) and scanned to identify SSRs by means of sputnik. Sequenced regions contained a total number of 211 SSRs distributed across the genome of the 16 target species with those of clade 7 (*P. alni, P. cambivora, P. europaea*, *P. fragariae *and *P. sojae*) and clade 8 (*P. lateralis *and *P. ramorum*) more highly represented (Fig. 3; Tables 3, 4, 5). A single microsatellite was identified in *P. inundata*. All SSRs identified in *P. infestans *were amplified with primers designed against its own genome (Fig. 3). Identified SSRs ranged in the number of repeats from 4 to 16, from 3 to 16 and from 4 to 14 in *P. sojae, P. ramorum *and *P. infestans *respectively (Tables 3, 4, 5). A single repeat of 24 was found in an SCRI isolate of *P. ramorum *(Table 4). Most SSRs were of seven repeats or less (88.9% *P. infestans*, 82.8% *P. ramorum*, 76.8 *P. sojae*), with a repeat number of four being the most common in all species (Fig. 4). Overall, the most common motifs were (AAG)n, (AGG)n and (AGC)n representing 40.9%, 23.3% and 17.6% respectively of the total number of identified SSRs (Fig. 5). Trinucleotide repeats were the most common (94.7%) followed by pentanucleotide (2.4%), tetranucleotide (1.9%) and dinucleotide (1.0%) repeats.

To evaluate intraspecific variability a few selected target regions amplified by primers S23F-S25R, S21F-S22R, I9-I10 and I5-6 (Table 2) were examined and sequenced from additional isolates of *P. alni*, *P. cambivora, P. cinnamomi*, *P. pseudosyringae *and *P. ilicis *(Table 1). The analysed target regions did not show intraspecific variability among analysed isolates of *P. alni *subsp. *alni*, *P. pseudosyringae *or *P. ilicis*, whereas *P. cambivora *and *P. cinnamomi *isolates were polymorphic in all the tested primer combinations. As an example, the target region amplified with primers I9-I10 from *P. cinnamomi *was characterised by 12, 14 and 18 repeated motifs (AGG) in three tested isolates (Table 6).

**Table 6:** Accession numbers and SSRs for selected microsatellites amplified and sequenced from two or more isolates of the same species to evaluate intraspecific variability.

***Phytophthora *species**	***Phytophthora *isolates**	**Primers**	**SSRs**	**Accession** **number**
***P. alni *subsp. *alni***	SCRP2	S23F-S25R	(agg)_7_; (aag)_10_; (agg)_16_; (aag)_12_; (aag)_4_	EF216565
	SCRP4		(agg)_7_; (aag)_10_; (agg)_16_; (aag)_12_; (aag)_4_	EF216567
	SCRP8		(agg)_7_; (aag)_10_; (agg)_16_; (aag)_12_; (aag)_4_	EF216566
				
***P. cambivora***	SCRP67	S23F-S25R	(agg)_10_; (aag)_10_; (agg)_9_; (aag)_12_; (agg)_4_; (agg)_7_	EF216569
	SCRP75		(agg)_9_; (aag)_10_; (agg)_11_; (aag)_12_; (agg)_6_	EF216571
	SCRP82		(agg)_10_; (aag)_10_; (agg)_9_; (aag)_12_; (agg)_5_	EF216570
				
***P. cambivora***	SCRP67	S21F-S22R	(agg)_4_; (aag)_5_; (aag)_4_	EF216581
	SCRP80		(agg)_4_; (aag)_5_; (aag)_4_	EF216582
	SCRP82		(aag)_4_; (agg)_6_; (agg)_4_	EF216583
				
***P. cambivora***	SCRP67	I9F-I10R	(acg)_4_; (aag)_4_; (agg)_6_	EF216516
	SCRP75		(acg)_4_; (aag)_4_; (agg)_6_	EF216519
	SCRP80		(acg)_4_; (aag)_4_; (agg)_6_	EF216517
	SCRP82		(aag)_5_; (agg)_5_	EF216518
				
***P. cinnamomi***	SCRP115	I9F-I10R	(aag)_14_	EF216509
	SCRP118		(aag)_18_	EF216511
	SCRP121		(aag)_12_	EF216510
				
***P. pseudosyringae***	SCRP674	I5F-I6R	(agc)_7_	EF216529
	SCRP734		(agc)_7_	EF216530
				
***P. ilicis***	SCRP377	I5F-I6R	(agc)_9_	EF216532
	SCRP379		(agc)_9_	EF216533

### Approach 2 – Identification of SSRs in *Phytophthora *spp. clade 7

The aim of this approach was to focus the search for SSR loci to a restricted range of four clade 7 *Phytophthora *species (*P. alni, P. cambivora, P. europaea and P. fragariae*) phylogenetically related to *P. sojae *(Fig 1B) [9].

#### Identification of target regions

This approach was based on a detailed list of SSRs identified in the genome of *P. sojae *and provided by Dr. Niklaus Grunwald at the Agricultural Research Service, U.S. Department of Agriculture, Corvallis, Oregon. Among the list, sixty genomic regions (500–1000 bp) were manually selected on the basis of having the longest SSRs in exons (20), introns (20) and non coding regions (20). The selected regions (Table 7) were screened using BLAST against the entire genomes of *P. ramorum *and *P. infestans *to search for homology irrespective of the SSR regions. None of these regions aligned with sequences from the *P. infestans *genome whereas 18 of the 60 regions were sufficiently conserved to match homologous genes in *P. ramorum *(6 were localised in exons and 12 in introns). Surprisingly none of these 18 regions contained SSRs in *P. ramorum*, however it was hypothesised that microsatellites could be present in homologous regions of other *Phytophthora *species more closely related to *P. sojae*. To verify this hypothesis, thirty-six primers (18 pairs) were designed in the conserved flanking regions and used to amplify the target regions from *P. alni, P. cambivora, P. europaea, P. fragariae *and an SCRI isolate of *P. sojae *(Table 7). Degenerate primers were designed when necessary.

**Table 7:** Set of primers designed with the 2^nd ^approach (Fig. 1B) to amplify genomic regions potentially containing SSRs in *Phytophthora *species of clade 7 [2].

**Forward primer**	**Reverse Primer**	**SSRs**	**Source^(a)^**	**Gene^(b)^**
S38F TCGTSTTCTACGTGCTGGAY	S39R GTAGCACGCGAACATGAASA	(AAC)_18_	scaffold_26: 667079–667377	E
S40F TTCCTTAAGTGGGGGAGGAT	S41R TRTCGGCRTTCAGCTTCTGT	(AAG)_4_; (AAG)_22_	scaffold_125: 153837–154222	I
S42F GCTGCAAGAGTCSCTCGAGTA	S43R CTTGAGGATGTCRATGAGCA	(AG)_21_	scaffold_89: 132819–133248	I
S44F GTRGCTCCTTCCTTAAGTGG	S45R GTGCTGCASGTAYGGCTTC	(AAG)_17_	scaffold_75: 344500–344901	I
S46F GTTGCGCGTGAGGTTCTC	S47R CAAAAGCTCTGCGTCC	(AG)_22_	scaffold_67: 282390–282656	I
S48F YCGGGCSACGGTAGG	S49R AAGAGCGTRAGCAGGAACC	(AG)_18_	scaffold_65: 225236–225440	I
S50F GTGGCTTCCACTGYTGCTG	S51R YATCAAGGACGTCAACTCGA	(AAC)_9_; (AACAGC)_23_	scaffold_48: 118994–119610	E
S52F CGGGATTTRTCRGATCAGG	S53R CTGTYTGATCARCTCTCCGCT	(AGG)_19_	scaffold_46: 153312–153646	E
S56F CACGAGCTGCAGKCATAYCT	S57R AGAATKGAMGCGATCGAC	(AGG)_16_	scaffold_21: 370731–371140	E
S58F TCGATCRACAGAAGCTGCWA	S59R GGAGTTCGCCATCAACAACT	(AAG)_14_	scaffold_19: 606139–606624	I
S60F GGCGTTTAAAGGCGTTTAAA	S61R CGTCTTCTTCTTGACGCACA	(AC)_18_	scaffold_52: 422559–422915	I
S64F YTTGCGACTAGCAAAGTGG	S65R CGAACTCCTTGTACAGGATGG	(AG)_14_	scaffold_56: 179585–179895	E
S66F GCAGYAGGCCCGGCCT	S67R GGAGTTCGCCATCAACAACT	(AAG)_11_	scaffold_12: 130593–130967	E
S68F CGTCGGTGGAGTAAACATCA	S69R AAAGGCGTTCGGAGAGYTG	(AG)_14_	scaffold_66: 83968:84423	I
S70F ATGACGAGGCAGCAGTTGAC	S71R AAGAACWGCGTSTACCTGCG	(ATC)_13_	scaffold_2: 564977–565285	I
S72F GCARCAATCTTCTGCTTYTTC	S73R ACACCTSCGTACWTTCGTCA	(AAC)_12_	scaffold_92: 221631–221935	I
S74F CGGTGGTACTTGTCGTCCTC	S75R TSTCCGGCTACATCATCATC	(ATT)_12_	scaffold_41: 327977–328190	I
S76F GCATCTACGACCAGATCTACCC	S77R GTAGACSGAGATGATGGCGT	(AC)_8_	scaffold_127: 112530–112930	I

^(a) ^= Gene sequences available at .

^(b) ^E = exons; I = Introns

#### Amplification, sequencing and SSR scoring

Most primer-species combinations produced single PCR bands of the expected size (Table 8). Purification and direct sequencing of these PCR fragments produced 54 reliable sequences which were analysed as previously described for Approach 1. Twelve different microsatellites were identified: 2 in *P. europaea*, 3 in *P. fragariae *and *P. alni *and 4 in *P. cambivora *(Table 8). Among these, 10 were trinucleotides and 2 were tetranucleotides repeated 4, 5 or 6 times. All regions sequenced from the SCRI isolate of *P. sojae *contained the predicted/expected SSR (Table 8).

**Table 8:** Accession numbers and SSRs for GenBank deposited sequences amplified using primers designed with the second approach (Fig. 1B).

**Selected** **primers^(a)^**	***Phytophthora *species**				
	
	***P. alni *subsp. alni** SCRP2	***P. cambivora*** SCRP67	***P. europaea*** SCRP622	***P. fragariae*** SCRP333	***P. sojae*** SCRP555
**S38–39**	EF382833 No SSR	EF382832 No SSR	EF382831 No SSR	EF382830 No SSR	EF382829 (aac)_18_
**S40–41**	NS^++^	EF382801 (aac)_4_	EF382800 No SSR	EF382799 (aac)_4_	NS^++^
**S42–43**	NS^++^	EF382798 (ACG)_6_	EF382797 (acg)_6_; (agg)_5_	EF382796 No SSR	EF382795 (ag)_21_
**S44–45**	EF382793 (aac)_4_	EF382792 (aac)_4_	EF382794 No SSR	NS^++^	EF382791 (aag)_5_; (aag)_5_
**S50–51**	EF382790 Any SSR	NA^+^	EF382789 No SSR	EF382788 No SSR	NS^++^
**S52–53**	NA^+^	EF382786 No SSR	EF382787 No SSR	NS^++^	EF382785 (agg)_19_
**S58–59**	EF382784 (aac)_4_	EF382783 (aac)_4_	EF382782 No SSR	EF382781 (aac)_4_	EF382780 (aag)_5_; (aag)_5_
**S64–65**	EF382828 No SSR	EF382827 No SSR	EF382826 No SSR	EF382825 No SSR	EF382824 (ag)_14_
**S68–69**	EF382820 (aagg)_4_	EF382821 No SSR	EF382822 No SSR	EF382831 (aac)_4_; (aagg)_4_	EF382819 (ag)_14_
**S70–71**	EF382815 No SSR	EF382816 No SSR	EF382817 No SSR	EF382818 No SSR	EF382814 (act)_13_
**S72–73**	EF382810 No SSR	EF382811 No SSR	EF382812 No SSR	EF382813 No SSR	EF382809 (aac)_12_
**S74–75**	NS^++^	NS^++^	EF382807 No SSR	EF382808 No SSR	EF382806 (aat)_12_
**S76–77**	EF382802 No SSR	EF382803 No SSR	NS^++^	EF382805 No SSR	EF382804 (ac)_8_

^(a) ^= Primers listed in Table 7 and not reported in this table are those that did not produce any reliable sequence.

NA^+ ^= Isolate-primer combinations that did not produce any amplification or produced complex profiles (two or more fragments).

NS^++ ^= Isolate-primer combinations for which single PCR bands were obtained but direct sequencing did not produced reliable results.

## Discussion

The present study was undertaken to develop a method to rapidly identify loci containing SSRs and to create a pool of microsatellite markers for species of the genus *Phytophthora *taking advantage of publicly available sequences for *P. sojae*, *P. ramorum *and *P. infestans*. Recently, Garnica et al. explored the transferability of microsatellites across *P. sojae*, *P. ramorum *and *P. infestans *via an *in silico *virtual PCR approach [27]. In the present study, such an analysis on the same three species was conducted but also followed up with a comprehensive screening and validation process on multiple species to provide a practical evaluation of the procedure as a means of accelerating the search for new SSR markers in the genus *Phytophthora*.

The first approach was aimed at the identification of informative SSR loci common to many *Phytophthora *species. This approach was based on the hypothesis that among the large number of microsatellites distributed across the genome of species of the genus there may be a proportion in genes common to many species with sufficient sequence conservation in flanking regions to allow the design and use of universal SSR primers. Our search of the predicted gene sets yielded approximately 10% fewer SSRs and a corresponding lower abundance of SSRs per Mb of sequence than that of Garnica et al [27]. Preliminary analyses revealed a very limited number of loci containing SSRs that were common to the three *Phytophthora *species tested. The majority of the identified loci (81.6%) were common to *P. sojae *and *P. ramorum *only which is consistent with their closer phylogenetic relationship in clades 7 and 8 than to *P. infestans *in clade 1 [9,2,3]. Similarly, Garnica et al. found in their *in silico *analysis that 7.5% of their primers were, in theory, transferable between at least two species (mainly *P. ramorum *and *P. sojae*) and only 1.0% transferable between the three species [27]. Among the selected sequences satisfying the above conditions, the number of repeats ranged from 3 to 17 and most SSRs showed seven repeats or less, with a repeat number of four being the most common in all species. The abundance of different repeat motifs differed slightly between species however, on average, (AAG)n, (AGG)n and (AGC)n were the most abundant triplets in all three Phytophthoras (Fig. 5). These results differ from those reported by Garnica et al. in which (AGC)n, (ACG)n and (AGG)n were the most abundant triplets amongst all the screened EST sequences [27]. It should, however be considered that unlike the study of Garnica our data are confined to SSR sequences for which it was possible to identify a homologue in at least one of the other two species. Therefore it could be hypothesised that motifs (AAG)n and (AGG)n are more abundant in more conserved genes. The dominance of trinucleotide SSRs compared to dinucleotide SSRs was not surprising considering that trinucleotides are abundant in coding regions of all higher eukaryotic genomes [31-33]. Dinucleotide repeats, in contrast, are characterised by higher mutation rates which may explain their abundance in introns and non-coding regions and lower frequency in coding regions, which cannot tolerate frame-shift mutations [34,35].

Primers designed in the present study with the first approach were tested against a panel of 16 different *Phytophthora *species representing the breadth of diversity across the genus to amplify *P. sojae*, *P. ramorum *and *P. infestans *target regions containing 33, 17 and 8 SSRs respectively. Overall, these primers enabled the sequencing of 171 target regions which contained 211 SSRs ranging in repeat number from 3 to 16. Most of these SSRs showed seven repeats or less with four the most common repeat number and (AAG)n, (AGG)n and (AGC)n the most common motifs. Trinucleotide repeats were dominant followed by pentanucleotide, tetranucleotide and dinucleotide repeats. This data indicate that such an approach can be useful to identify cross-specific SSR loci in the genus *Phytophthora*. As further genome sequences become available, for example, *P. capsici *, the process can be refined to specific subsets of the genus. The mutation rates and, consequently, the practical utility of the identified SSRs in the study of the specific *Phytophthora *species need to be examined further. Undoubtedly, a risk of this approach is that the selection is biased towards more conserved sequences which may subsequently have a lower mutation rate that reduces their utility as polymorphic markers. Furthermore, the fact that *P. infestans *SSRs were all identified using primers designed on its own genome (Fig. 3) may indicate that this approach is less appropriate for distant relatives considering that, as stated above, *P. infestans *is phylogenetically distant from *P. sojae *and *P. ramorum*. However, the identification of intraspecific polymorphisms in some selected SSRs is encouraging and demonstrates that at least some of the selected SSRs are valuable for immediate practical applications (Table 6). This is consistent with the reported applicability of EST-SSRs across closely related taxa in other organisms as well as *Phytophthora *[23,36-38]. In the present study, the focus on the breadth of species (16) prevented the analyses of a wider number of target regions. However, the same method could be easily applied to the study of more regions from one or a few species.

The application of the first method enabled the identification of novel SSRs from all the 16 target species with those of clade 7 and 8 more highly represented (Fig. 3). A higher proportion of SSRs from species of the clade 7 and 8 was expected considering that *P. sojae *and *P. ramorum *belong to these two clades [2,3]. In light of this fact, a second approach to identify a greater number of polymorphic SSRs from within a more limited range of clade 7 taxa more closely related to *P. sojae *was investigated. Sixty *P. sojae *SSR candidates were compared by BLAST analysis against the complete genome sequence of the other two species yielding 18 SSR candidates which could be aligned with homologous regions in *P. ramorum*. However in none of these 18 candidates (6 exons and 12 introns) was the SSR maintained in *P. ramorum*. In four of the more closely related species (*P. alni, P. cambivora, P. europaea *and *P. fragariae*), however, some of the SSR regions were conserved (Table 8). In this study the focus was on discovery of SSRs in invasive forest *Phytophthora *species within the clade 7a, perhaps a higher success rate in marker discovery would have followed a search amongst the closest related species in clade 7b (*P. sinensis*, *P. melonis*, *P. cajanae *and *P. vignae*) [2]. Although a few SSR markers with potential were discovered using this approach, it was not a highly efficient means of identifying new polymorphic SSR loci and highlights the lack of conservation of SSR loci, even amongst coding regions within a single ITS clade of *Phytophthora*. Some degree of cross-species amplification has been observed between SSRs in *P. infestans *with other Clade 1c taxa and it is therefore likely that a wider application of this method concentrated on the closest relatives would be more productive [23].

## Conclusion

The present study has tested two different methods to generate SSR markers that can be utilised across a broad range of *Phytophthora *species. The final number of identified loci for any single species may not be sufficient to run a complete population genetics analysis and key studies on the inter- and intraspecific variation remain. A comprehensive dataset of candidate SSRs from a range of species has been created (Table 3, 4, 5). The detailed groundwork needed to amplify these regions from such a diverse collection of species and target regions has been completed which moves beyond the previous *in silico *approach to improve our understanding of the range and sequence conservation of SSR loci amongst species [27]. In general, the level of interspecific SSR sequence conservation, even amongst more closely related species within a single clade, was low and the method may not be the most efficient means of identifying novel SSR loci. Apart from their application as molecular markers, determining the abundance and density of SSRs in Oomycetes may help understand whether these sequences have any functional and evolutionary significance [17]. Furthermore, irrespective of the microsatellites, some of the amplified regions represent valuable marker regions for a number of applications [39]. A single optimal target gene for all *Phytophthora *species and assay requirements is unlikely to exist, therefore the continued identification and characterization of new target genes offers new opportunities for detection and phylogenetic studies [3,40,41].

## Methods

### *Phytophthora *isolates and DNA extractions

Twenty-eight isolates (16 *Phytophthora *species) sourced from the SCRI culture collection were used in this study (Table 1). Isolates and species were selected to represent taxa most relevant to European forestry that also represented the breadth of *Phytophthora *diversity defined according to clades based on ITS sequence analysis [2]. Isolates were stored on oatmeal agar at 5°C and grown on French bean agar for routine stock cultures.

Total DNA was extracted from pure cultures of *Phytophthora *according to Schena and Cooke, diluted to 10 ng/μl and maintained at 5°C for routine amplifications and at -20°C for long term storage [42].

### Analysis of sequences from *P. infestans*, *P. sojae *and *P. ramorum *genome projects and scanning for homologous SSRs

The predicted protein datasets of *P. infestans *(from the NCGR XGI database that was available prior to the Broad genome sequencing project) and *P. ramorum *and *P. sojae * were screened for SSR loci using Sputnik (Chris Abaijan ). Pairwise BLAST analysis using the default parameters was used to select loci conserved in different species combinations [43]. Manual screening of these loci on the basis of SSR and flanking region DNA sequence conservation yielded a short-list for further analysis.

### Primer design and amplification conditions

All primers (Table 2 and 7) were designed with the Primer3 Software set up to generate a Tm of 60°C ± 2, a GC% between 20 and 80% and a length of 18–26 bp [44]. Primers were purchased from Eurogentec ltd. (Belgium). Considerable effort was made to obtain successful amplification of single PCR bands from as many species as possible. This involved adjustment of MgCl_2 _concentration (0.7, 1.0 or 1.7 mM) and annealing temperatures (55 or 58°C) for PCR reactions (Table 3, 4, 5). Furthermore in some circumstances alternative primers were designed and tested to amplify the target regions from as many taxa as possible (Table 2). PCR reactions were performed in a total volume of 15 μl containing 10 ng of genomic DNA, 1.5 μl of 10× Reaction Buffer (Promega Corporation, WI, USA), 100 μM dNTPs, 0.7, 1 or 1.7 mM MgCl_2_, 15 μg BSA, 2 unit of *Taq *polymerase (Taq DNA polymerase, Promega Corporation) and 1 μM of primers. PCR amplification conditions consisted of: 1 cycle of 95°C for 2 min; 40 cycles of 94°C for 30 s, 55 or 58°C for 30 s, 72°C for 60 s; and a final cycle of 72°C for 5 min.

### DNA sequencing

The best primers and amplification conditions were identified for all primer-species combinations and target DNA was re-amplified in a total volume of 50 μl to provide sufficient amplicon for direct sequencing. Single PCR bands were purified with the MinElute PCR Purification Kit (Qiagen Ltd. West Sussex, UK) to remove excess primers and nucleotides. Sequencing was carried out with the same primers utilized for the amplification in a dye-terminator cycle-sequencing reaction (FS sequencing kit, Applied Biosystems, Warrington, UK) and run on an ABI373 automated sequencer (Applied Biosystems). All selected PCR fragments were sequenced using both the forward and the reverse primers.

### Sequence analysis and SSRs scanning

The "Sequence Navigator" software (Applied Biosystems) was utilised to evaluate reliability of sequences and to compare forward and reverse sequences to create a consensus sequence. Non-reliable sequences in which both forward and reverse sequences contained doubtful bases were discarded. All sequences obtained in the present study were also parsed to a web version of SPUTNIK , which uses a recursive algorithm to search for repeated patterns of nucleotides of length between 2 and 5.

## Authors' contributions

LS conceived the study, carried out the molecular analyses, optimized the described method, performed data acquisition, data analysis and data interpretation and wrote the manuscript.

LM performed analysis of sequences from genome projects and scanned for homologous SSRs.

DELC coordinated, supervised and contributed to the design of the study, provided intellectual input to optimize the method and revised the manuscript critically.
